# Nanomagnesium promotes moso bamboo tolerance to cadmium toxicity: insights from photosynthesis phenomics, oxidative metabolism, and cellular adjustments

**DOI:** 10.3389/fpls.2025.1636755

**Published:** 2025-08-13

**Authors:** Aamir Mehmood Shah, Zaid Ulhassan, Yi Peng, Cong Ma, Xinyu Du, Muhammad Iftikhar, Mohamed S. Sheteiwy, Ali El-Keblawy, Di Li, Qibing Chen, Shiliang Liu

**Affiliations:** ^1^ College of Landscape Architecture, Sichuan Agricultural University, Chengdu, Sichuan, China; ^2^ School of Breeding and Multiplication (Sanya Institute of Breeding and Multiplication), Hainan University, Sanya, Hainan, China; ^3^ Department of Integrative Agriculture, College of Agriculture and Veterinary Medicine, United Arab Emirates University, Al-Ain, United Arab Emirates; ^4^ Department of Applied Biology, College of Sciences, University of Sharjah, Sharjah, United Arab Emirates; ^5^ Geophysical Exploration Brigade of Hubei Geological Bureau, Wuhan, Hubei, China

**Keywords:** MgONPs, nanotechnology, moso bamboo tolerance, metal stress, cadmium

## Abstract

Cadmium (Cd) stress severely hampers plant growth in forest ecosystems. Although magnesium oxide nanoparticles (MgONPs) are known to reduce Cd toxicity in numerous plant species, their detoxification mechanisms in Moso bamboo (*Phyllostachys edulis*) remain unexplored. The present study investigates how MgONPs mitigate the Cd-induced phytotoxic effects in *P. edulis* by examining morpho-physiological and cellular oxidative repair mechanisms. Results revealed that MgONPs diminished the oxidative stress by reducing hydrogen peroxide (26/21%), superoxide radical (29/23%), and malondialdehyde (25/20%) contents in leaves/roots under Cd stress. Moreover, MgONPs improved the photosynthetic performance as revealed by higher chlorophyll and gas exchange levels, correlated with better growth and biomass, under Cd stress. Interestingly, MgONPs improved the plant defense by escalating the activities of antioxidant enzymes (ascorbate peroxidase, catalase, and superoxide dismutase) and metabolites (total phenolics, flavonoids, tocopherols) accumulation. Importantly, anatomical analyses verified MgONPs’ role in repairing Cd-induced distortion to stomatal aperture, guard cells integrity and ultrastructural damages. These outcomes demonstrate the MgONPs application greatly enhanced the bamboo tolerance to Cd toxicity by simultaneously regulating the photosynthetic efficiency, multiple antioxidant defense mechanisms, recovering cell damages, and restricting Cd-accumulation. This study provides bamboo-specific mechanistic insights in advancing the understanding of nanoparticles assisted phytoremediation in woody perennials.

## 1 Introduction

Moso bamboo (*Phyllostachys edulis*), a prominent member of Gramineae family, covers more than five million hectares of forests in China, dominating more than 70% of country’s bamboo-growing regions and significantly contributing to its economy and ecology (Wang et al., 2016; Zhao et al., 2018). Owing to its rapid growth rate and elevated biomass production, this plant species presents a promising candidate for enhanced pollutant bioaccumulation and phytoremediation applications (Ahmad et al., 2021; Kumar et al., 2021; Emamverdian et al., 2021). Soil contamination with heavy metals has greatly threatened the sustainable development of agricultural forestland, especially in south-west region of China (Wang et al., 2019). Cadmium (Cd) contamination is a widespread abiotic stressor that significantly impacts plant productivity (Yağcı et al., 2024; He et al., 2025; Gao et al., 2025). Cd can readily accumulate within the terrestrial and aquatic environments. This leads to the transfer of trace metals, eventually into human bodies *via* food chain (Qiu et al., 2021). The major sources of soil Cd pollution include industrial activities such as uncontrolled release of their wastes, mining, electroplating industry and other anthropogenic activities (Zou et al., 2021). An excessive accretion of Cd in the tissues of various plant species severely hampered the bamboo growth performance, inhibits photosynthetic capacity, uptake of nutrients, destroyed the cellular homeostasis, regulated the oxidative stress metabolism, disrupted the important metabolic processes and ultrastructural abnormalities, ultimately leading to compromised the development of plants (Li et al., 2016; Emamverdian et al., 2021, 2022; 2023a, b). Therefore, scientific community is primarily concerned with Cd toxicity, emphasizing the pivotal steps to limit the Cd bioavailability in plants.

The use of nanotechnology, particularly in the form of nanoparticles (NPs), is a rapidly growing field with significant potential for applications such as nanomaterials and nano fertilizers in agricultural areas (Ulhassan et al., 2025; Meng et al., 2025). Their small sizes, easily water-solubility, economic feasibility, and high efficiency for absorption by plants make them suitable candidate for potential utilizations in agriculture and environment fields (Mittal et al., 2020). Different metal or metallic oxide NPs have been reported to confer bamboo resilience against metals toxicity as well as facilitate plant growth (Emamverdian et al., 2022, 2024). It has been reported that particular concentrations of zinc nanoparticles, iron oxide nanoparticles, titanium oxide and silicon oxide nanoparticles effectively improved the bamboo tolerance to copper, Cd, arsenic, and lead stresses by decreasing metals accumulation, oxidative stress, peroxidation of lipids, improving chlorophyll contents, soluble proteins level, antioxidant defense system and recovering cell damages in bamboo (*Arundinaria pygmaea*) (Emamverdian et al., 2021, 2023a, 2024). In other plant species, recent studies reported that differently applied MgONPs enhanced the Cd tolerance by improving growth and biomass, photosynthesis, antioxidant enzyme activities and reducing Cd accumulation in wheat (*Triticum aestivum*) (Cheng et al., 2020), tobacco (*Nicotiana tabacum*) (Qin et al., 2025), and spinach (*Spinacia oleracea*) (Taj et al., 2024). However, the mechanisms by which MgONPs enhance Cd tolerance may vary depending on plant species, growth conditions, and nanoparticle properties. The novelty of the current study lies in the fact that there are no reports on the potential beneficial roles of MgONPs in regulating the accumulation and adverse effects of Cd in *P. edulis*.

Thus, a detailed analysis at morphological, metabolic, biochemical, and cellular levels is required to fully comprehend the mechanisms behind the beneficial mechanisms of MgONPs in *P. edulis* plants under Cd stress conditions. For this study, we targeted plant growth performance (shoot height, root length, dry and fresh weight), endogenous accumulation of Cd contents, oxidative stress markers, photosynthetic attributes, metabolites production, nutrients accumulation, enzymatic based antioxidant defense mechanisms, and changes in anatomical structures. Assessing these study biomarkers will be beneficial for cultivating bamboo plants in Cd-contaminated soils and advancing the development of MgONPs as sustainable and efficient nano-fertilizers in agriculture.

## 2 Materials and methods

### 2.1 Characterization of nanoparticles

MgONPs were commercially obtained from Cw-Nano (www.cwnano.com), according to manufacturer specifications, the purity of the MgONPs sample was 99.9% and their particle sizes range from 20 to 30 nm. The transmission electron microscope (TEM) was used to evaluate the shape and size of the MgONPs using a JEOL JEM-1230 apparatus (JEOL Ltd., Akishima, Japan). The scanning electron microscope (SEM) was used to evaluate the size distribution of NPs using a Hitachi TM-1000 instrument (Hitachi Ltd., Tokyo, Japan). Additionally, the elemental composition and abundance of Mg and O in MgONPs specimen was examined utilizing an energy-dispersive X-ray spectroscopy and detector-equipped field emission scanning electron microscope (Ali et al., 2024).

### 2.2 Soil pot experiment

A pot experiment was carried out from December 2023 to May 2024. The experimental soil was collected from the top 0–20 cm layer of a field near our research site. The soil was removed of impurities, blend homogenized, air-dried, and passed through a 5.0 mm sieve before use. Prior to the experiment, its key chemical and physical properties were assessed, with results showing a pH of 6.7, organic matter of 60.1 g kg^–1^, available nitrogen (N) of 218 mg kg^–1^, phosphorus (P) 12.5 mg kg^–1^, and potassium (K) contents of 85.2 mg kg^–1^, respectively. Once air-dried, a portion of the airdried soils was carefully blended with Cd: (i) CK: control, soil free of Cd and MgONPs, (ii) only NPs: soil free of Cd, with plants receiving a foliar spray of 50 µM MgONPs, (iii) NPs+Cd: soil amended with 120 mg kg^–1^ of Cd, with plants receiving a foliar spray of 50 µM MgONPs, (iv) Cd: soil amended with 120 mg kg^–1^ of Cd, without MgONPs application. The applied Cd concentration (120 mg kg^–1^) in soil was selected from Li et al. (2016), while 50 µM MgONPs concentration was determined via preliminary trials to ensure efficacy without causing phytotoxicity. The mixtures were then left to equilibrate for 21 days in a shaded condition. In December 2023, the studied bamboo plants around 1.0 m in height and 1.5 cm in diameter were carefully selected from a one-year-old bamboo field in Anji County, Zhejiang, China. Subsequently, the seedlings were transferred into sixteen plastic pots containing 5 kg of prepared soil (**Table 1**). These potted plants were then placed randomly in plastic trays and positioned within a greenhouse at the landscape ecological base of Sichuan Agricultural University, Chengdu, China. Tap water was supplied as irrigation during the experiment to account for water loss and maintain the soil at 75% of its field water-holding capacity. The exogenous application of 50 µM MgONPs was foliar-sprayed after 15 days of plant transfer. This practice was repeated three times during the whole experiment with intervals of 33 days. All the bamboo plants were maintained in the greenhouse with these conditions; natural light, 70/80% as day/night humidity level, and 25/30°C as day/night temperature.

**Table 1:** Physical and chemical properties of soil samples before spiking with Cd.

Soil properties	Units	Values
Class of soil texture		Loam
sand	%	40
clay	%	11
silt	%	49
soil pH	/	6.7
soil organic matter	g kg^–1^	60.1
available nitrogen	mg kg^–1^	209
available phosphorus	mg kg^–1^	12.5
available potassium	mg kg^–1^	81.2
total Cd	mg kg^–1^	0.17

### 2.3 Measurement of morphological attributes

After five months of soil Cd exposure (by mid-May 2024), all treated bamboo plants were harvested. Roots were cleaned with distilled water, then soaked in 20 mM Na_2_-EDTA for 15–20 minutes to remove surface-bound metals. Subsequently, plants were rinsed three times with deionized water. Root and shoot length were assessed with the help of a centimeter measuring scale. Stems, roots, and leaves were separated to record their morph-physiological, biochemical, and ultrastructural studies. The separated plant parts were oven-dried at 65°C for about 48 hours before measuring their dry weights.

### 2.4 Pigment contents, gas exchange parameters and fluorescence parameters

The fully expanded leaves were selected for spectrophotometric quantification of chlorophylls contents and carotenoids as described previously (Lichtenthaler and Buschmann, 2001). Leaf gas exchange measurements including stomatal conductance, photosynthetic rate, transpiration rate, and intercellular CO_2_ levels were quantified using a Li-Cor 6400 portable photosynthesis system (Li-Cor Inc., Lincoln, NE, USA) between 7 am to 10 am (Ulhassan et al., 2023). The fluorometer was used to measure maximal chlorophyll fluorescence emission (Ulhassan et al., 2023). In details, the *Fv/Fm* (maximum quantum efficiency of photosystem II) was concluded by first adapting the second set of fully extended leaves in darkness for 20 minutes, and then fluorescence imaging measurements were conducted using a pulse-amplitude-modulation fluorimeter (IMAG-MAXI, Heinz Walz GmbH, Effeltrich, Germany).

### 2.5 Estimation of nutrients accumulation and endogenous Cd contents

To estimate Cd and macronutrients (N, Mg, and Ca), root and leaf samples (0.3 g each) were oven-dried for 48 hours, grounded into powder, and then ashed at 500 °C for 12 hours. A 0.2 g portion of the powdered sample was digested at 80 °C using a tri-acid mixture consisting of 1 mL H_2_SO_4_, 5 mL HNO_3_, and 1 mL HClO_4_, until a transparent solution was obtained. After air-cooling, the final digested solution was diluted to 50 mL with deionized water, filtered, and then examined for elemental concentrations via atomic absorption spectrometer (AA6300, SHIMADZU, Kyoto, Japan) (Ulhassan et al., 2023).

### 2.6 Determination of H_2_O_2_, O_2_^•-^ and MDA

For the estimation of hydrogen peroxide (H_2_O_2_), leaves or roots (0.5 g each) samples were recovered in an ice bath using 0.1% w/v tri-chloric acid, homogenize then centrifuged for 15 minutes at 15,000 *g.* Subsequently, 0.5 mL of the obtained supernatant was mixed with 0.5 mL of 50 mM potassium phosphate buffer and 1 mL of 1 M potassium iodide solution. The absorbance was measured at 390 nm and standard curve was used to determine the H_2_O_2_ levels (Velikova et al., 2000). The superoxide radicals (O_2_^•–^) contents of plant tissues were determined using established method (Jiang and Zhang, 2002). The malondialdehyde (MDA) contents were determined according to earlier protocol (Heath and Packer, 1968).

### 2.7 Observation of cellular structures and assays of antioxidants enzyme activities

The preparation of leaf samples for SEM and TEM analysis was done by adapting the same procedure (Ulhassan et al., 2023). Leaf and root samples weighing approximately 0.5 g were ground in 50 mM K_3_PO_4_ buffer and centrifuged at 12,000 *g* for 15 minutes. The obtained supernatant was employed in antioxidant enzymes analysis. Superoxide dismutase (SOD) and peroxidase (POD) activities were calculated spectrophotometrically at 560 nm and 470 nm, respectively as reported (Zhang et al., 2008). Additionally, activities of catalase (CAT) (Aebi, 1984), ascorbate peroxidase (APX) (Nakano and Asada, 1981), and glutathione reductase (GR) (Jiang and Zhang, 2002) were determined using standard protocols.

### 2.8 Determination of metabolites

For the quantification of total phenolic and total flavonoids, leaf and root samples were ground into fine powder using mortar and pestle in the presence of 80% ethanol. The ethanolic extractions were stopped as the pellet become colorless and then centrifuged at 4000 g for 10 min and supernatants were stored until further use. The estimation of total phenolic content was performed with Colin-Ciocalteau method (using sodium carbonate as reagent). The absorbance of reaction mixture was observed at 650 nm (Makkar et al., 1993). The total flavonoids content in leaf and root extracts was estimated spectrophotometrically by measuring absorbance at 430 nm (Djeridane et al., 2006). The method of Li et al. (2013) was employed to measure the tocopherol content. In this experiment, 0.1 g of leaf tissue was extracted with 3 mL of ethanol and centrifuged (10,000 × g for 10 minutes). The resulting supernatant (0.1 mL) was mixed with 1 mM phosphoric acid, 0.2 mL of 0.2% bathophenanthroline, and 0.001 M ferric chloride. Absorbance was recorded at 534 nm, and tocopherol concentration was determined using a tocopherol acetate (TAC) standard curve, expressed as mM tocopherol acetate equivalents per gram of plant material.

### 2.9 Statistical analysis

All data analyses and statistical evaluations were measured using SPSS Statistics version 20 software platform (IBM Corporation, USA). The statistical analysis involved conducting a one-way analysis of variance (ANOVA), followed by Duncan’s multiple range test, to identify significant differences among the treatment group means (*n*=4 plants per treatment) at a significance level of *p ≤* 0.05. All results are presented as mean ± standard deviations (SDs) of 4 biological replicates. The graphs and principal component analysis (PCA) were generated using the Origin Pro version 8.0 (Origin Lab Corporation, Wellesley Hills, Wellesley, MA, USA).

## 3 Results and discussion

### 3.1 Characterization of MgONPs

**Figure 1:**
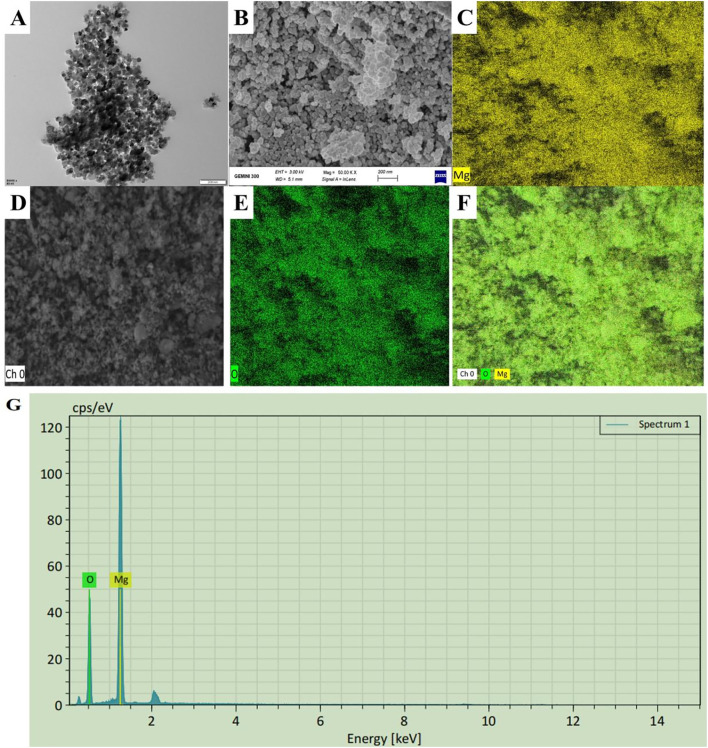
Characterization of MgONPs. **(A)** SEM (scale bar= 100 nm) image **(B, C)** EDX (scale bar= 500 nm) spectra, **(D–G)** elemental composition of MgONPs illustrating their morphology.

The particle sizes of MgONPs were analyzed using the TEM images, which revealed a spherical shape with an average size of 32 nm (**Figure 1A**). This spherical structure and the particle size range of 25–40 nm were clearly matched with previous studies (Ali et al., 2024). Whereas, the SEM images displayed a uniform arrangement of NPS with minimal agglomeration (**Figure 1B**), potentially caused by evaporation during drying. The energy dispersive X-Ray spectroscopy (EDX) microanalysis confirmed the chemical composition of the samples, showing prominent peaks for magnesium (Mg) and oxygen (O) in the spectra (**Figures 1C–G**). The EDX mapping validated these results, clearly indicating that Mg and O were the primary components in the NP samples. The absence of peaks for other elements confirmed the purity of the samples. Previous studies have also reported Mg and O as the dominant elements in biologically synthesized MgONPs, as evidenced by EDS spectra (Ali et al., 2024). These findings confirm the high purity of the MgONPs used in this study.

### 3.2 Effects of MgONP on bamboo growth performance under Cd stress

**Figure 2:**
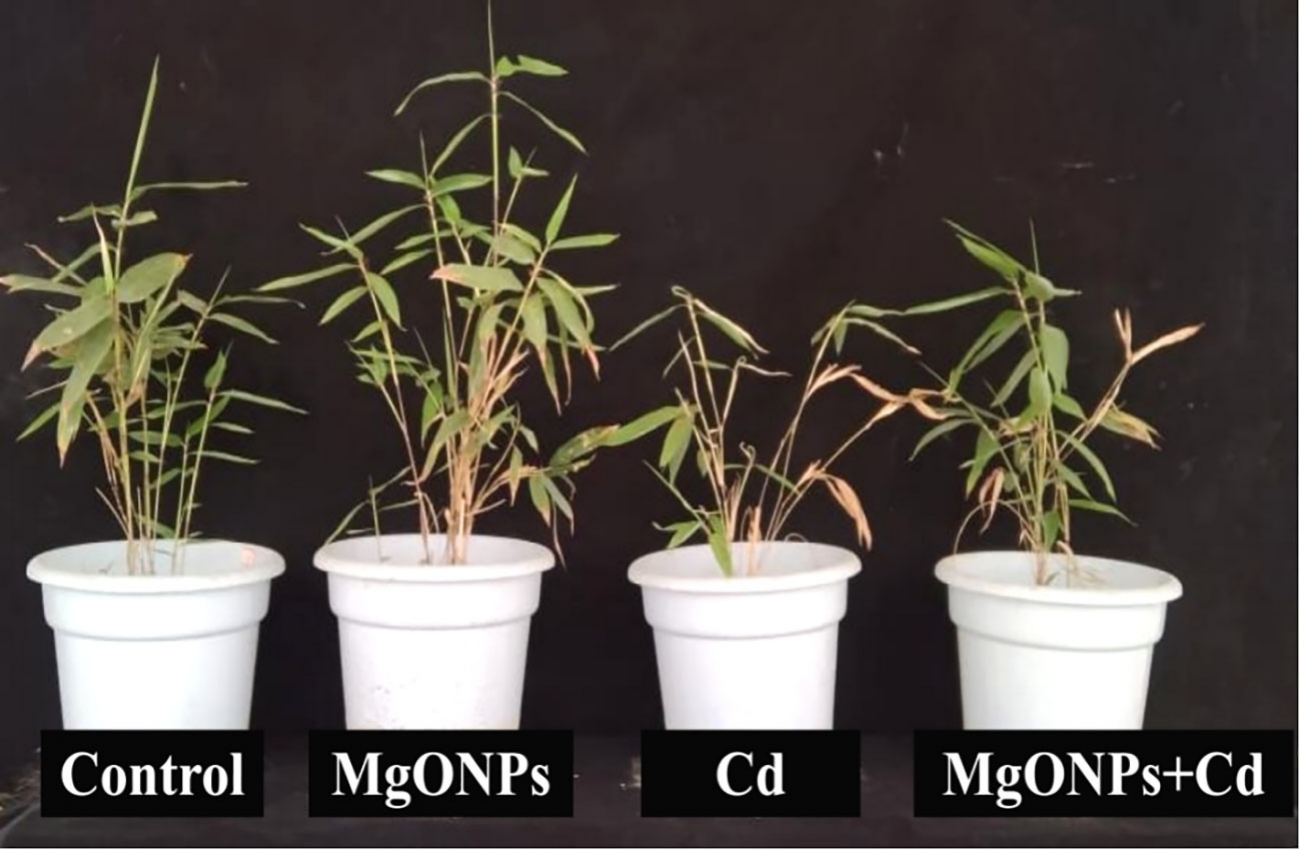
Plant phenotypes under control, MgONPs, Cd, and MgONPs+Cd treatments.

**Figure 3:**
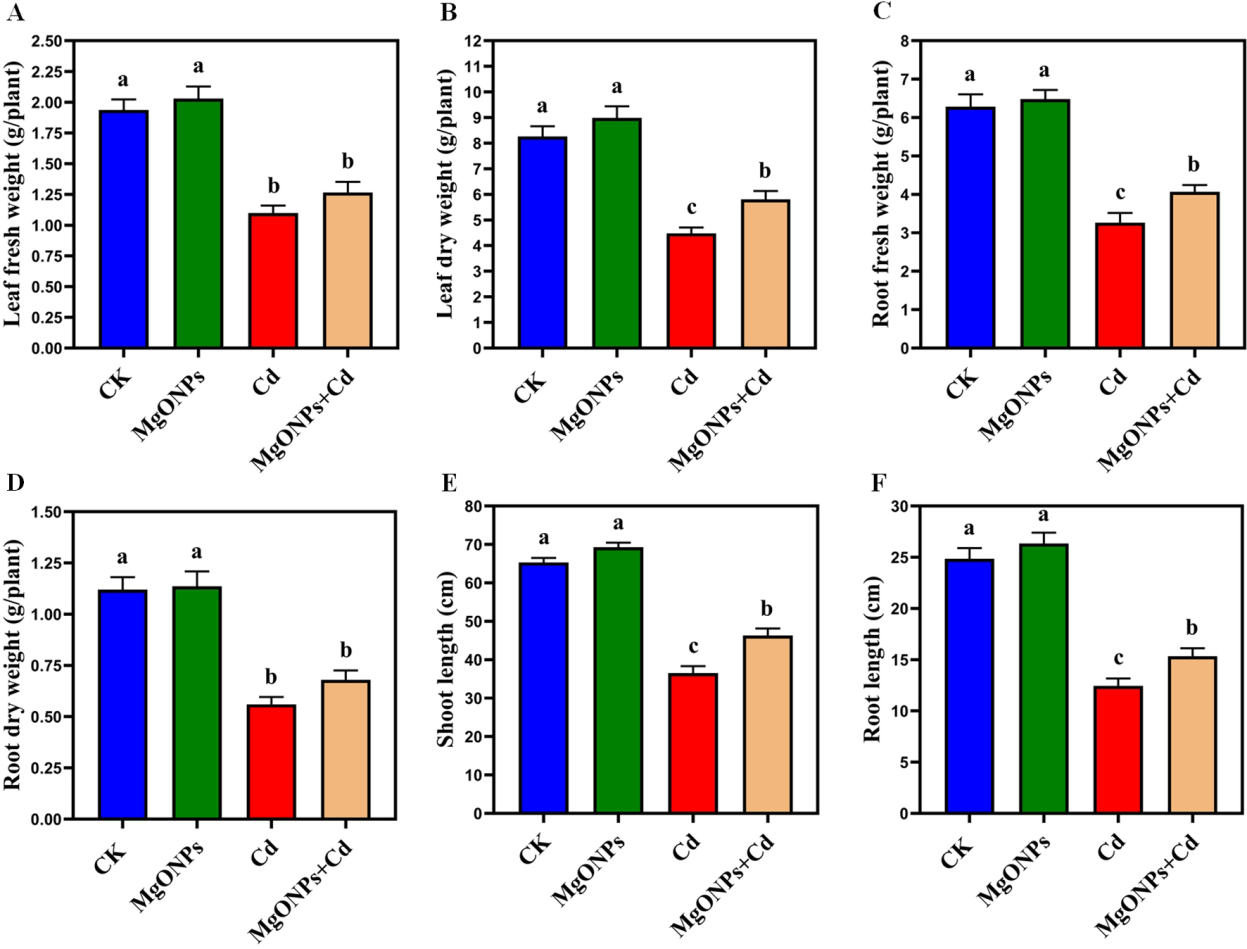
Effects of MgONPs on the growth performance of moso bamboo plants with and without Cd stress. **(A)** leaf fresh weight **(B)** leaf dry weight **(C)** root fresh weight **(D)** root dry weight **(E)** shoot length **(F)** root length. The data show the mean values of four replicates (n = 4 ± SD). Different letters denote statistically significant differences according to Tukey’s test at *p* ≤ 0.05.

**Figure 4:**
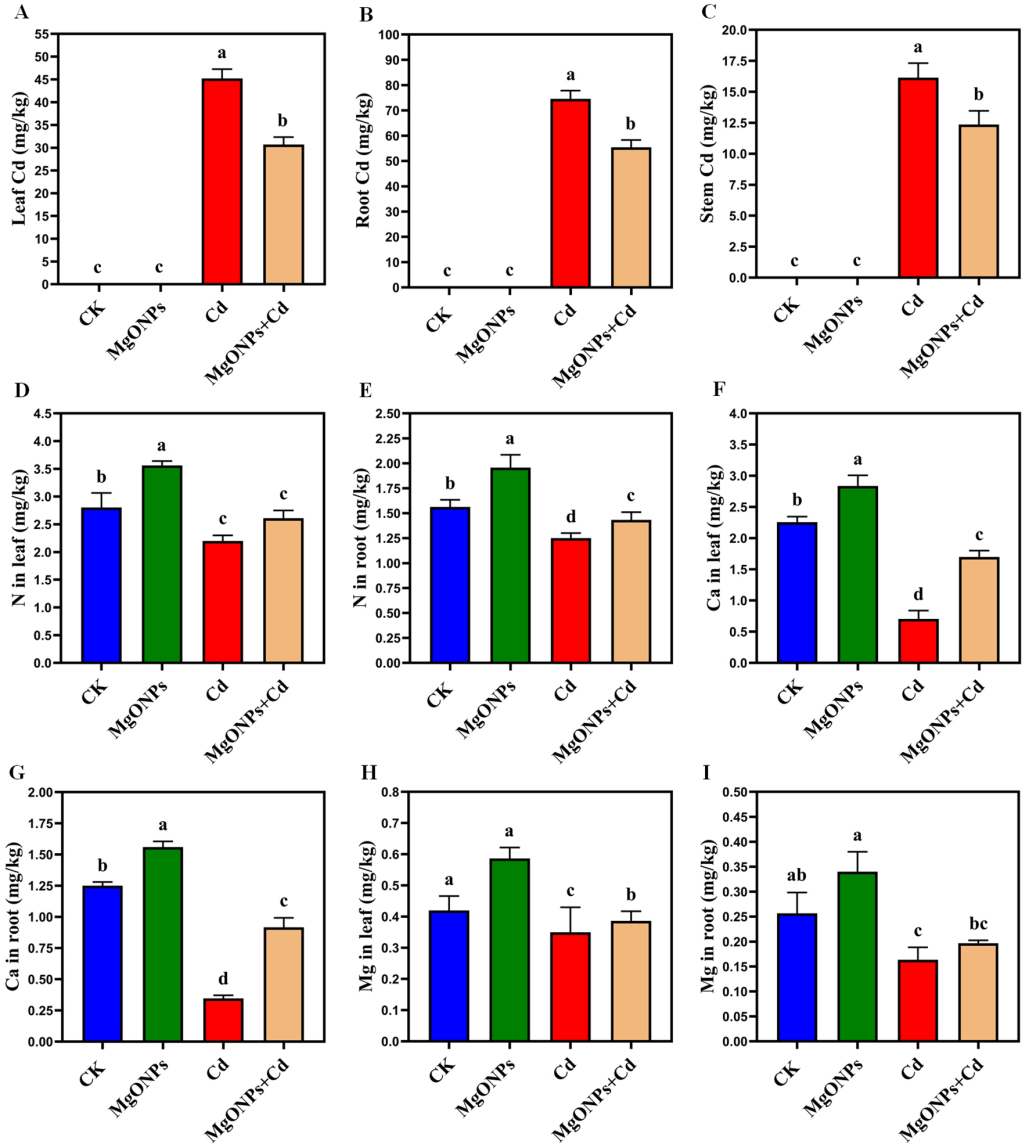
Effects of MgONPs on the endogenous cadmium and nutritional profile of moso bamboo plants. **(A)** Cd in leaves **(B)** Cd in roots **(C)** Cd in stems **(D)** N (nitrogen) in leaves **(E)** N in roots **(F)** Ca (calcium) in leaves **(G)** Ca in roots **(H)** Mg (magnesium) in leaves **(I)** Mg in roots. The data show the mean values of four replicates (n = 4 ± SD). Different letters denote statistically significant differences according to Tukey’s test at *p* ≤ 0.05.

**Figure 5:**
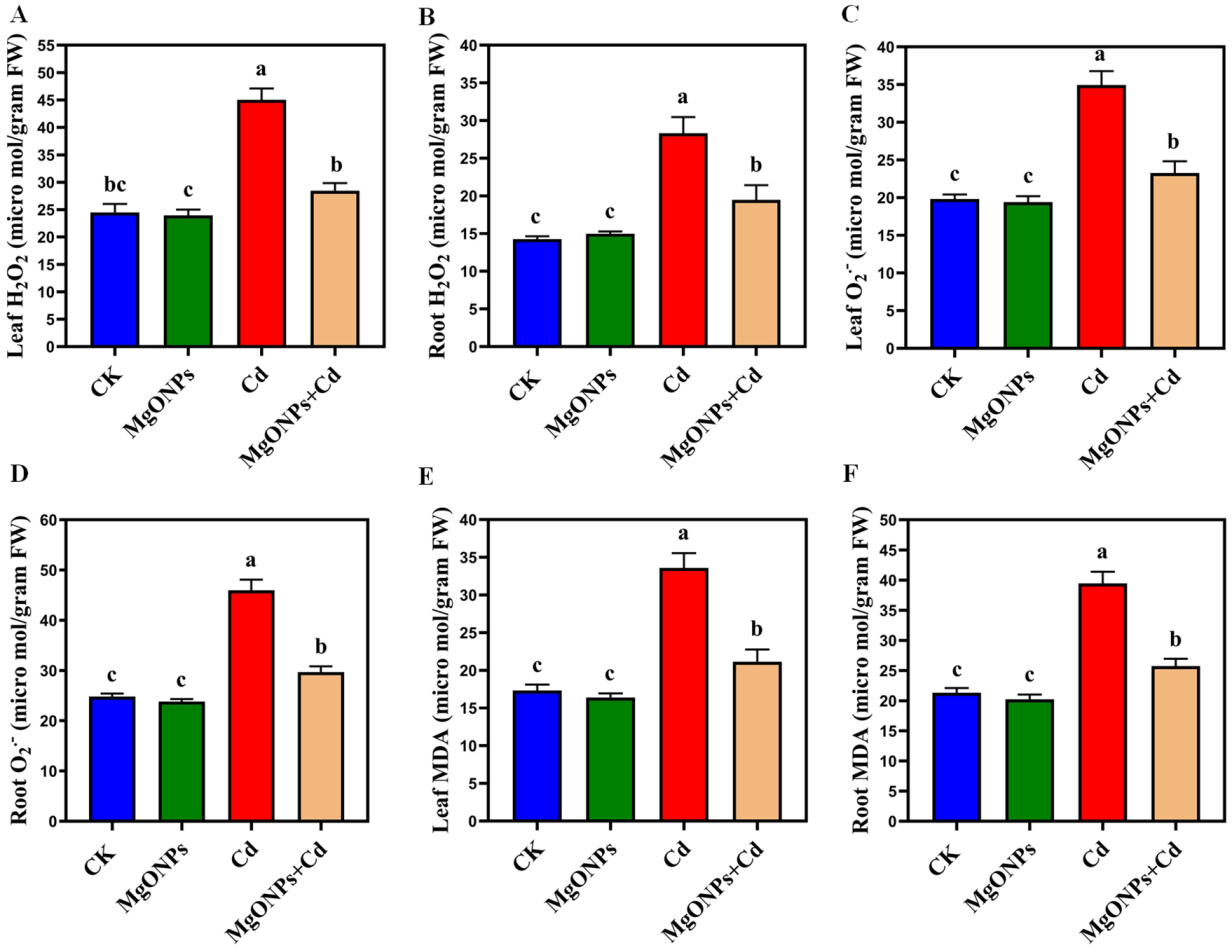
Effects of MgONPs on the accumulation and visualization of reactive oxygen species of moso bamboo plants treated with and without Cd stress. **(A)** H_2_O_2_ in leaves, **(B)** H_2_O_2_ in roots, **(C)** O_2_^•–^ in leaves, **(D)** O_2_^•–^ in roots, **(E)** MDA in leaves, **(F)** MDA in roots. The data show the mean values of four replicates (n = 4 ± SD). Different letters denote statistically significant differences according to Tukey’s test at *p* ≤ 0.05.

**Figure 6:**
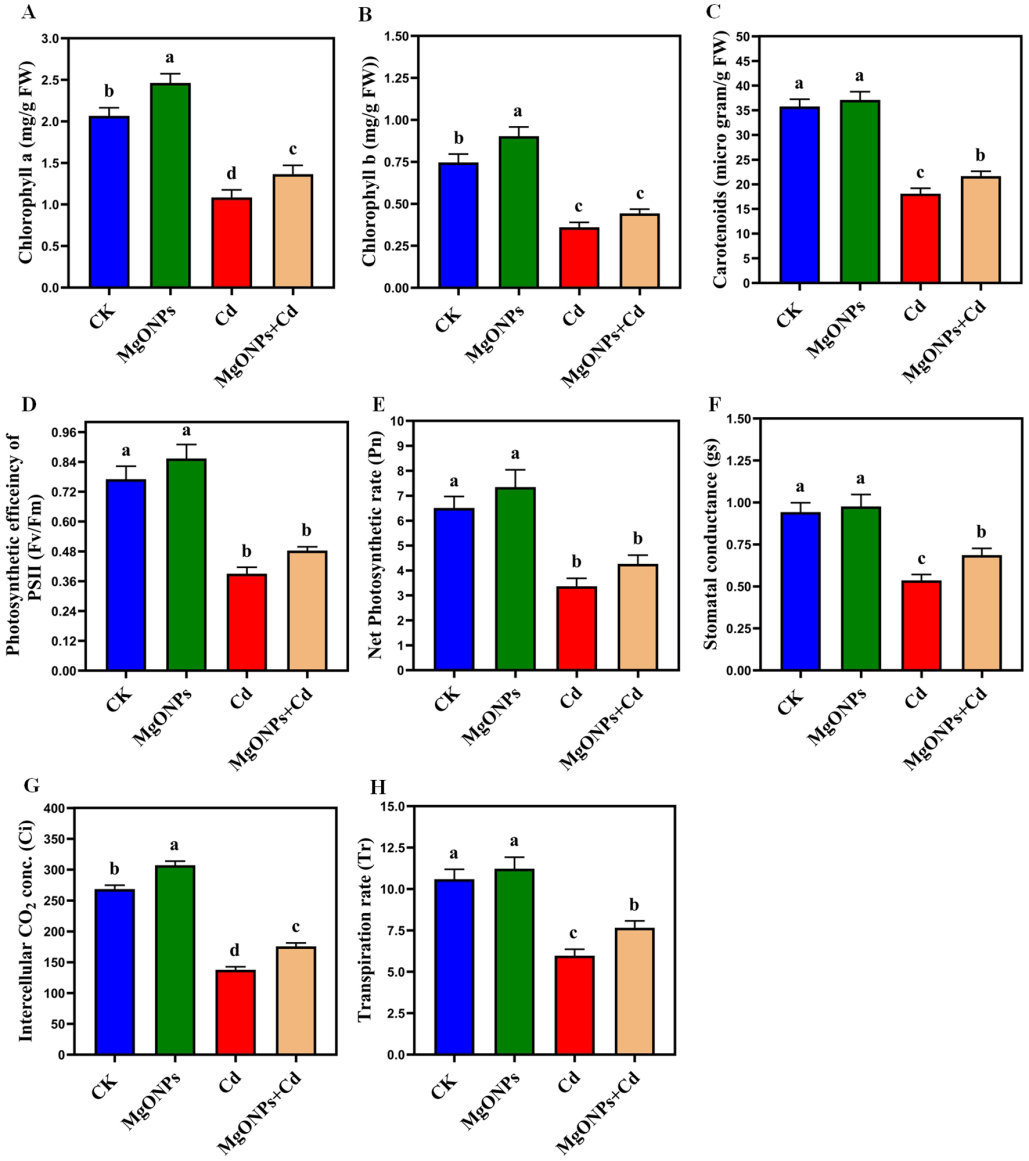
Effects of MgONPs on the photosynthetic attributes of moso bamboo plants with and without Cd stress. **(A)** Chlorophyll a **(B)** chlorophyll b **(C)** carotenoids **(D)** photosynthetic efficiency of PSII **(E)** net photosynthetic rate **(F)** stomatal conductance **(G)** intercellular CO_2_ conc. **(H)** transpiration rate. The data show the mean values of four replicates (n = 4 ± SD). Different letters denote statistically significant differences according to Tukey’s test at *p* ≤ 0.05.

**Figure 7:**
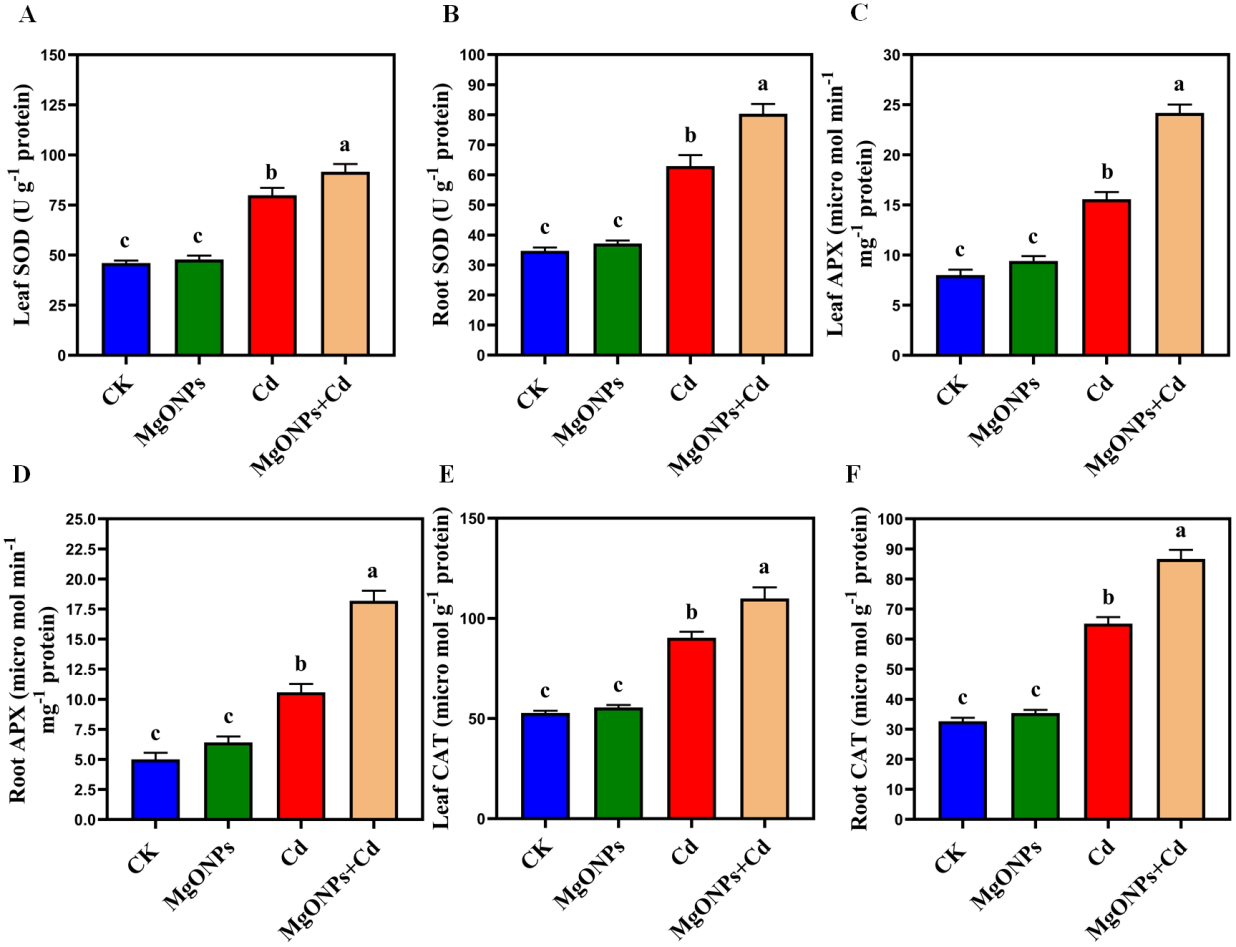
Effects of MgONPs on antioxidants enzyme activities of moso bamboo plants treated with and without Cd stress. **(A)** SOD (superoxide dismutase) in leaves **(B)** SOD in roots **(C)** APX (ascorbate peroxidase) in leaves **(D)** APX in roots **(E)** CAT (catalase) in leaves **(F)** CAT in roots. The data show the mean values of four replicates (n = 4 ± SD). Different letters denote statistically significant differences according to Tukey’s test at *p* ≤ 0.05.

Phenotypic images clearly showed the changes in growth and biomass under control, MgONPs, Cd and MgONPs + Cd treatments (**Figure 2**). Compared to control bamboo plants, the application of MgONPs alone treatments marginally increased the leaf fresh weight (LFW), leaf dry weight (LDW), root fresh weight (RFW), root dry weight (RDW), shoot length (SL), and root length (RL). In contrast, Cd exposure alone significantly reduced these growth parameters, with decreases of 44% in SL, 47% in LFW, 42% in LDW, 48% in RFW, 45% in RDW, and 49% in RL compared to the control. While, the combined application (MgONPs+Cd) effectively mitigated Cd-induced toxicity, leading to improvements in SL (26%), LFW (23%), LDW (18%), RFW (25%), RDW (21%), and RL (23%) relative to the Cd-only treatment (**Figures 3A–F**). The reduced bamboo growth and biomass under Cd stress may be attributed to excessive Cd accumulation (**Figures 4A-C**), higher reactive oxygen species (ROS) levels (**Figures 5A–D**) and inhibition in nutrients uptake (**Figures 4D–I**) within tissues. Moreover, excess Cd accumulation may hinder the absorption and transport of nutrients and water, disrupting photosynthesis and resulting in decreased growth and biomass of bamboo shoots and roots (Qadir et al., 2013). Few studies have reported that Cd exposure diminished the bamboo and other plant growth and biomass (Zulfiqar et al., 2022; Emamverdian et al., 2022; Behtash et al., 2024). Furthermore, alone exposure of MgONPs promoted the plant growth and biomasses (**Figures 2**, **3A–F**). The enhancing effects of multiple NPs on plant growth are well-established (Daler et al., 2024; Ali et al., 2024; He et al., 2025). Under Cd exposure, MgONPs substantially improved the growth and biomass which could be associated with the reduction in Cd bioaccumulation (**Figures 4A–C**), decrease in cellular oxidative damage (**Figures 4D–F**), enhancement in photosynthetic potential (**Figures 6A–H**), balanced nutrients level (**Figures 4D–I**) and reinforcement of the antioxidant mechanisms (**Figures 7A–F**). A positive correlation between NPs application, and improved plant growth and biomass was associated with better nutrients uptake, decreased oxidative stress, and reduced Cd bioaccumulation as reported in bamboo plants (Tafazoli et al., 2017; Emamverdian et al., 2021, 2023b).

### 3.3 MgONPs modulated photosynthesis phenomics in response to Cd stress

With the supply of only MgONPs, there was a little increase in the levels of Chla, Chlb, carotenoids, Fv/Fm, Tr, Gs, Pn, and Ci in comparison to their respective controls. Relative to the control, plants exposure to Cd stress drastically diminished the Chla (47%), Chlb (51%), carotenoids (49%), Fv/Fm (48%), Pn (52%), gs (47%), Ci (50%) and Tr (48%). Under Cd stress, MgONPs improved the Chl a (27%), Chl b (31%), carotenoids (24%), Fv/Fm (25%), Pn (28%), Gs (23%), Ci (26%) and Tr (28%) (**Figures 6A–H**). The enhancement in photosynthetic pigments and gas exchanges by MgONPs alone supply could be attributed to increased leaf Mg content (**Figure 4H**), higher respiration and elevated photosynthetic capacity (Ali et al., 2024). The increase in Fv/Fm observed with MgONPs suggests an enhanced rate of electron transport. Thus, MgONPs can assist plants in mitigating reactive oxygen species in PSII and enhancing its functionality. The decrease in pigments caused by Cd stress could be linked to distortions in chloroplast structures (Yang et al., 2021) and inhibition of chlorophyll synthesis that disrupt the production of leaf pigments. The decrease in Fv/Fm under Cd stress can lower the net photosynthetic rate and impair electron transport between PSII and PSI, ultimately affecting the pigments production (He et al., 2025). MgONPs treatments significantly counteracted the aforementioned photosynthetic suppression and reinstated the photosynthetic characteristics under Cd stress (**Figures 6A–H**). Earlier studies reported that different NPs improved the pigment contents and photosynthetic efficiency in Cd-stressed bamboo plants (Emamverdian et al., 2021; Taj et al., 2024).

### 3.4 MgONPs minimizes Cd-accumulation and regulates nutrients accumulation under Cd stress

To assess the possible impacts of MgONPs on intrinsic Cd and nutrients accumulation, we evaluated their concentrations in leaf/root tissues (**Figures 4A–I**). It was observed that alone Cd treatments significantly increased endogenous Cd levels to 45.21 mg kg^–1^ in leaves, 16.14 mg kg^–1^ in stems, and 74.56 mg kg^–1^ in roots. In Cd-stressed moso bamboo plants, MgONPs treatments notably reduced the Cd accumulation in leaves (32%), stems (23%), and roots (26%). The higher Cd accumulation in roots compared to stems and leaves indicates the roots’ strong capacity to retain Cd, effectively limiting its transport to aerial parts (Ulhassan et al., 2025). This observation aligns with previous studies reporting higher Cd concentrations in roots than leaves (Kaur et al., 2024). Whereas, pretreatment with MgONPs exhibited a “nano-effect”, significantly decreasing Cd accumulation by restricting its movement from roots to upper plant parts. The lower Cd translocation factor indicates that MgONPs efficiently limit the movement of Cd from roots to shoots, thereby decreasing Cd accumulation in leaves. The antagonistic effects between Mg and Cd, and the regulation in the transcript levels of Cd transporter genes largely participated in reducing the Cd-accumulation as reported in recent study. The mitigating effect of MgONPs likely involves two key mechanisms: (1) ionic antagonism, where Mg^2+^ ions released from MgONP dissolution compete with Cd^2+^ for uptake transporters (e.g., NRAMP1 and ZIP family proteins) on root cell membranes, thereby reducing Cd absorption; and (2) enhanced binding site density in root cell walls, particularly carboxyl groups on pectin molecules, which immobilize Cd^2+^ in the apoplast and limit its symplastic transport to shoots. These mechanisms collectively contribute to the observed reduction in Cd accumulation by MgONPs (Qin et al., 2025). The alone MgONPs treatments considerably enhanced nutrient levels in Cd-exposed moso bamboo plants. Compared to unexposed control plants, MgONPs alone increased N (26/23%), Ca (21/18%), and Mg (18/14%) contents in leaves and roots, respectively. Yet, Cd exposure severely disrupted ionic homeostasis, with a significant decrease in N (51/48%), Ca (57/50%), and Mg (51/48%) levels compared to non-Cd-exposure plants. Under MgONPs + Cd, MgONPs improved the levels of Mg (39/34%), Ca (32/28%), and N (19/25%) contents in leaves and roots. These results suggest that MgONPs enhance nutrient uptake by mitigating Cd-induced inhibitory effects on nutrient transport. The beneficial impacts of MgONPs on nutrients transport were correlated to plant biomass production (**Figures 2**, **3A–F**), photosynthetic performance (**Figures 6A–H**) and antioxidants defense system (**Figures 7A-F**). The excessive Cd accumulation and its structural similarity to essential elements (Mg and Fe) may explain its role in inhibiting the nutrient uptake (Handa et al., 2019). Matched findings have been reported in earlier studies that different NPs enhance the nutrients availability in bamboo and other plants, thereby assisting in alleviating the heavy metals toxicity (Daler et al., 2024; Emamverdian et al., 2023a).

### 3.5 MgONPs minimized the oxidative stress indicators under Cd stress

To appraise the impact of MgONPs on Cd-induced oxidative stress, we estimated MDA, H_2_O_2_ and O_2_^•–^ contents in the leaves/roots (**Figures 5A–F**). Compared to the control group, Cd-alone treatment significantly increased the accumulation of H_2_O_2_ (83% in leaves and 98% in roots), O_2_^•–^ (76% in leaves and 85% in roots), and MDA (93% in leaves and 85% in roots). Higher accumulation of these oxidative markers was observed in roots compared to leaves, indicating more severe oxidative stress in roots. Cd exposure excessively elevated H_2_O_2_ and O_2_^•–^ levels, leading to a further rise in MDA levels in moso bamboo tissues (**Figures 5A–F**). Possibly, Cd renders extra ROS, causing higher MDA, undermines membrane integrity and cellular organization (Emamverdian et al., 2023b, 2024). In contrast, MgONPs treatment effectively mitigated Cd-induced oxidative damage by reducing the overproduction of H_2_O_2_ (26/21%), O_2_^•–^ (29/23%), and MDA (25/20%) contents in leaves or roots. This suggests that MgONPs reduce the lipids peroxidation, oxidative stress and maintain the integrity of cellular membrane by limiting the ROS and MDA generation caused by Cd in moso bamboo tissues. Previous reports support our observations that MgONPs minimized the oxidative damages and maintained the plasma membrane integrity in bamboo species under heavy metal stress (Emamverdian et al., 2024). Similarly, Kaya et al. (2025) demonstrated that long-term soil management and organic amendments improved berry biochemistry in grapes, offering a comparable perspective on sustained physiological improvement under abiotic stress.

### 3.6 MgONPs resists stomatal closure and cellular ultrastructural damages

**Figure 8:**
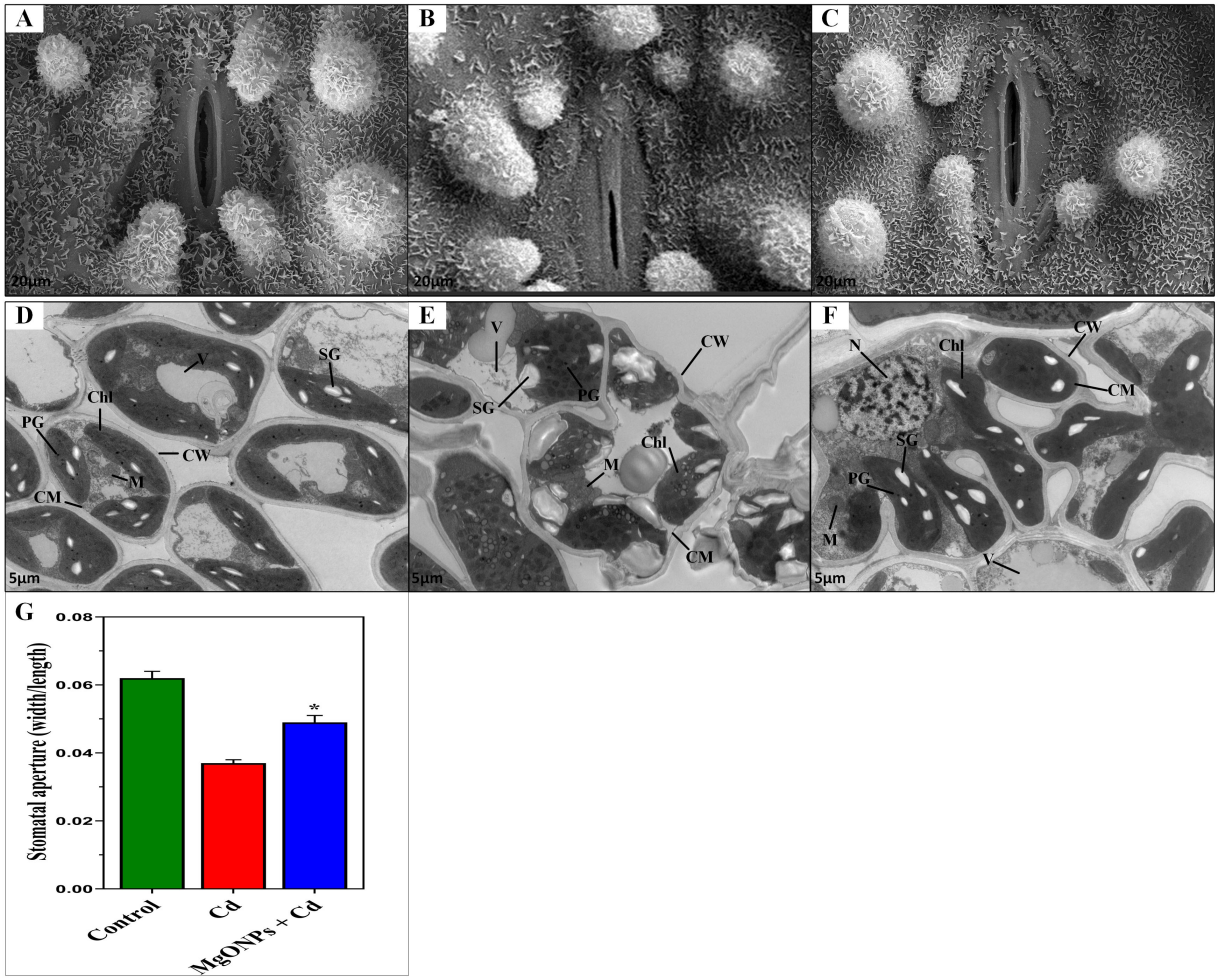
Effects of MgONPs on the leaf stomata opening and cellular ultrastructure of moso bamboo plants treated with and without Cd stress. Leaf stomata opening; **(A)** displays the control treatment under no-stressed normal conditions, **(B)** displays disruption in stomata guard cell and stomatal aperture under Cd stress, **(C)** displays the recovery in stomata damages under Cd+ MgONPs treatments. Leaf ultrastructural changes; **(D)** shows control, non-stressed normal conditions, **(E)** shows severe ultrastructural damages under Cd stress alone, **(F)** displays recovery in cell damages under MgONPs + Cd treatment. Cell wall (CW), cell membrane (CM), mitochondria (M), chloroplast (Chl), vacuole (V), plastoglobuli (PG), starch grain (SG), and nucleus (N), and **(G)** stomatal aperture.

To examine the potential effects of MgONPs in protecting the guard cells and stomata shape, stomata were observed on leaf epidermis (**Figures 8A–C**). In non-stressed untreated control plants, a well-defined epidermal structure with regularly spaced stomata with a smooth waxy cuticle surrounding the stomatal opening was noticed (**Figure 8A**). Under Cd stress, disrupted stomatal structure, a deformation in epidermal cells, and deposition of crystalline particles on the leaf surface was observed (**Figure 8B**). On the contrary, the exogenous MgONPs significantly reversed the Cd-induced damages to guard cells and stomata shape (**Figure 8C**), showing the crucial role of MgONPs in the protection of guard cells from oxidative damages induced by Cd stress. These findings suggest that optimal levels of MgONPs may regulate the function of stomatal pores via certain mechanisms, including electrochemical and hydraulic adjustments in guard cells and the regulation of osmotic pressure. These processes might be associated with the capacity of MgONPs to eliminate H_2_O_2_ and decrease lipid peroxidation by boosting antioxidant activity (Emamverdian et al., 2022).

The ultrastructures of leaf mesophyll cells after exposure to Cd and/or MgONPs were displayed (**Figures 8D-F**). Under control (non-stress) conditions, leaves exhibited a mature mitochondrion (M), well-defined cell wall (CW), cell membrane (CM), organized clusters of small plastoglobuli (PG), along with seeable and ample-sized starch grains (SG) (**Figures 8D**). In contrast, Cd-alone treatment caused severe structural damage, including scattered and swollen CW and CM, deformed vacuoles (VAC), disrupted PG, and a ruptured nucleus (N) with nucleolus (Nue) were observed. Additionally, granum thylakoids (GT) in chloroplasts were disorganized, starch grains (SG) appeared irregular, and chloroplasts (Chl) showed abnormal shapes (**Figure 8E**). The MgONPs+Cd treatments significantly reduced Cd-induced cellular damage, as evidenced by a well-developed, thick CW and CM, mature M, oval-shaped Chl, organized small-sized PG, regular shape of starch grain (SG), large-sized VAC, and large size of stomatal aperture (**Figure 8F, G**). These results suggest that Cd stress causes ultrastructural damage in leaf cells, likely due to excessive ROS generation, resulting in oxidative cellular damage, as previously reported (Li et al., 2016; He et al., 2025). In contrast, MgONPs recuperates the Cd-mediated cellular distortions. The probable cause is that MgONPs reduce the excessive ROS generation induced by Cd in cellular organelles, effectively rescuing cellular damage by maintaining cell integrity, and cell structures, and ensuring membrane stability. Consistent with our results, a new study revealed that MgONPs reduced the ultrastructural damages caused by Cd stress in leaves (Cheng et al., 2020).

### 3.7 MgONPs regulated the antioxidants defense system under Cd stress

To gain deeper insight into the free radical scavenging activities of MgONPs against Cd-elicited oxidative damage, we evaluated the antioxidant defense system (**Figures 7A–F**). The application of MgONPs alone resulted in a slight increase in SOD, APX, and CAT enzyme activities in both leaves and roots compared to the control. Since, MgONPs function primarily as a slow-release source of Mg^2+^ ions to support essential physiological processes without inducing oxidative stress, their standalone application does not trigger significant upregulation of antioxidant defense enzymes (Ali et al., 2024). Under Cd-alone treatments, a significant rise in the activities of antioxidant enzymes, including SOD (73% in leaves and 81% in roots), APX (94% in leaves and 111% in roots), and CAT (71% in leaves and 99% in roots), was observed. The inclusion of MgONPs further increased the activities of these enzymes in both leaves and roots under Cd stress. The Cd+MgONPs combination significantly increased the antioxidant enzyme activities because Cd triggered oxidative stress (via extra ROS production) that demand urgent antioxidant upregulation. This suggests MgONPs play displayed synergistic effects with antioxidant defense pathways, effectively scavenging Cd-induced oxidative stress, scavenged free radicals production, preserved the membrane integrity and enhanced the Cd-tolerance in moso bamboo. Earlier studies also supported that different NPs escalated the antioxidant enzymes to enhance the heavy metals tolerance in various bamboo species (Emamverdian et al., 2022, 2024). Nonetheless, the response of antioxidant enzymes to MgONPs is intricate and can differ with the type of target bamboo specie, and exposure method or time duration of heavy metals.

### 3.8 MgONPs improved metabolites accumulation under Cd stress

**Figure 9:**
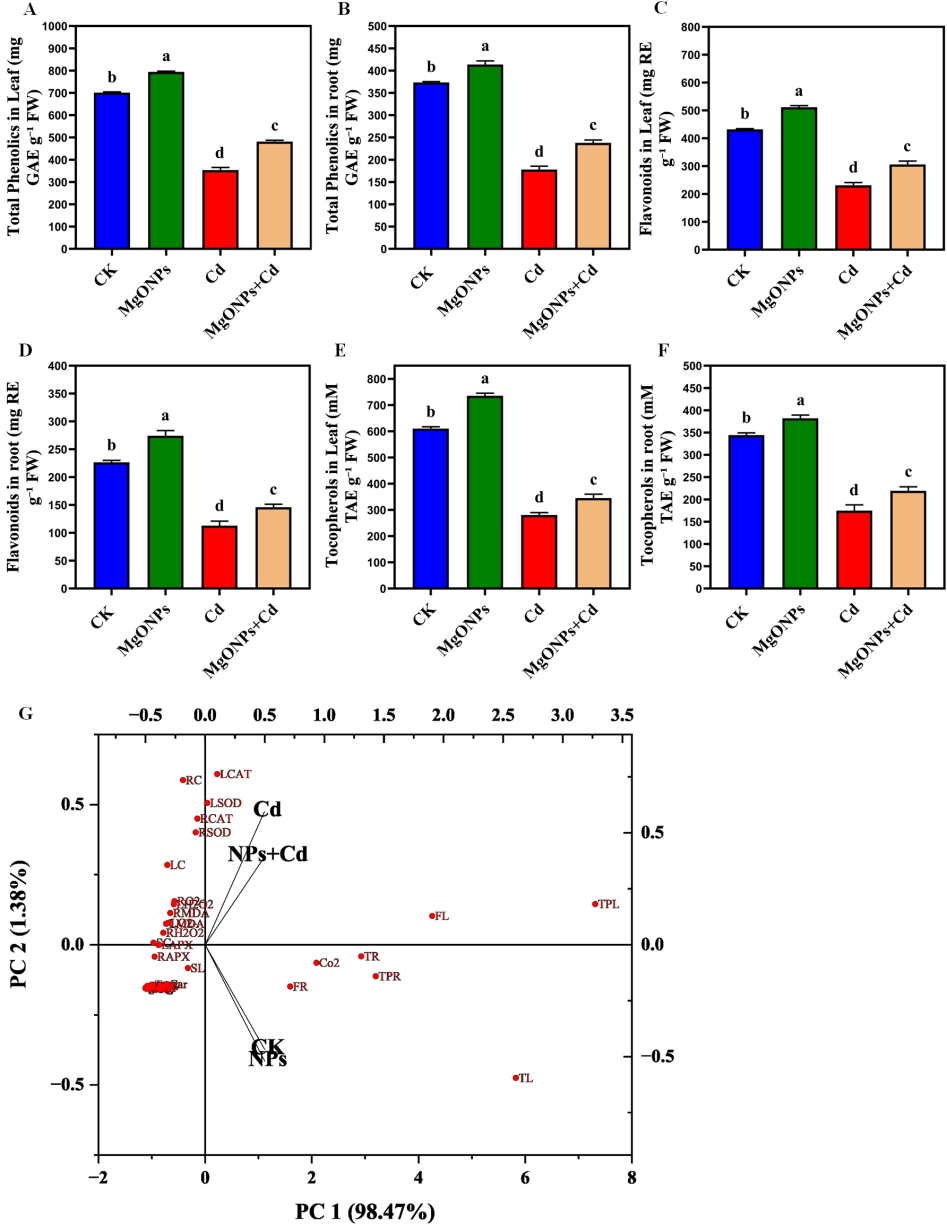
Effects of MgONPs on the metabolites of moso bamboo plants treated with and without Cd stress. **(A)** total phenolics in leaves **(B)** total phenolics in roots **(C)** flavonols in leaves **(D)** flavonols in roots **(E)** tocopherols in leaves **(F)** tocopherols in roots. The data show the mean values of four replicates (n = 4 ± SD). Different letters denote statistically significant differences according to Tukey’s test at *p* ≤ 0.05. **(G)** Principal component analysis (PCA) plot illustrating the grouping of treatments based on selected variables. Red dots represent individual variables, while black arrows indicate the direction and distribution of treatment groups (CK, MgONPs, Cd, Cd+MgONPs). The separation and direction of groups highlight their differential impacts on the analyzed traits (Shoot length (SL), root length (RL), leaf fresh weight (LFW), leaf dry weight (LDW), root fresh weight (RFW), root dry weight (RDW), leaf Cd (LC), root Cd (RC), stem Cd (SC), chlorophyll a (*Chl a*), chlorophyll b (*Chl b*), carotenoids (Car), net photosynthetic rate (NPR), photosynthetic efficiency PSII (*Pn*), stomatal conductance (*gs*), Intercellular CO_2_ conc. (CO_2_), transpiration rate (*Tr*), shoot H_2_O_2_ (LH_2_O_2_), root H_2_O_2_ (RH_2_O_2_), leaf O_2_ (LO_2_), root O_2_ (RO_2_), shoot MDA (SMDA), root MDA (RMDA), Leaf SOD (LSOD), root SOD (RSOD), leaf CAT (LCAT), root CAT (RCAT), leaf APX (LAPX), root APX (RAPX), leaf Mg (LMg), root Mg (RMg), leaf Ca (LCa), root Ca (RCa), leaf N (LN), root N (RN), total phenolics in leaf (TPL), total phenolics in root (TPR), flavonols in leaf (FL), flavonols in root (FR), tocopherols in leaf (TL), and tocopherols in root (TR)).

Phenolic compounds containing carboxylic groups are known for their roles as scavengers of free radicals and derive the production of antioxidant enzymes (Ali-Arab et al., 2022; Ulhassan et al., 2025). The total phenolics, flavonoids, tocopherol were marginally enhanced in response to MgONPs alone treatments in leaves/roots relative to the control treatment (**Figures 9A–F**). While, Cd alone supplementation drastically reduced the total phenolics (47/52%), flavonoids (46/51%), tocopherols (53/49%) in leaves and roots in comparison to their control group. The reduced biosynthesis of total phenolics, flavonoids, tocopherols compounds under Cd stress conditions indicates the inhibitory effects of Cd stress on their production. The reduced production of these phenolic compounds by Cd alone stress was also observed in earlier studies (Emamverdian et al., 2022, 2023b). Whereas, MgONPs + Cd treatments palliated the toxic effects of Cd as manifested from the improvement in total phenolics (34/29%), flavonoids (32/28%), tocopherol (22/25%) in leaves/roots comparison to their respective Cd alone treatments (**Figures 9A–F**). Under heavy metals stress conditions, phenolic compounds oxidize the ROS compounds and provide protection to bamboo plants (Emamverdian et al., 2024). There might be a link between the concentration of Mg and the carboxyl and hydroxyl groups found in phenolic compounds. It has been shown that phenols can decrease superoxide production from the Fenton reaction through the release of chelating Fe or Mg ions (Rezayian et al., 2023). Previous research has stated that different NPs enhanced the production of these phenolic compounds under heavy metals stress conditions (Qi et al., 2021; Emamverdian et al., 2024; Ulhassan et al., 2025).

### 3.9 Multivariate data analysis distinguished the effects of MgONPs applications on bamboo plants under Cd toxicity

The PCA biplot illustrates that Cd exposure markedly altered the physiological and biochemical responses of bamboo plants. The two principal components, PC1 (98.47%) and PC2 (1.38%), together explain more than 99% of the total variance, capturing nearly the entire variability in the dataset. In the plot, red dots represent individual physiological and biochemical variables (e.g., antioxidant enzyme activities, oxidative stress markers, and growth traits), while black vectors indicate the treatment groups. The Cd-treated samples cluster on the left side of the plot and are closely associated with oxidative stress-related variables such as H_2_O_2_, MDA, SOD, and CAT, suggesting elevated oxidative damage under Cd stress. Conversely, the CK (control) and NPs (MgONPs-only) treatments appear on the opposite side, reflecting improved physiological performance, including better growth and reduced oxidative stress. The NPs+Cd treatment is positioned between Cd and CK/NPs, indicating a moderate recovery effect, likely due to the protective role of MgONPs in mitigating Cd toxicity. Overall, this PCA analysis distinctly separates the treatment responses and highlights the beneficial role of MgONPs in alleviating Cd-induced stress by enhancing antioxidant defenses and promoting nutrient uptake and plant growth (**Figure 9G**).

## 4 Conclusion

**Figure 10:**
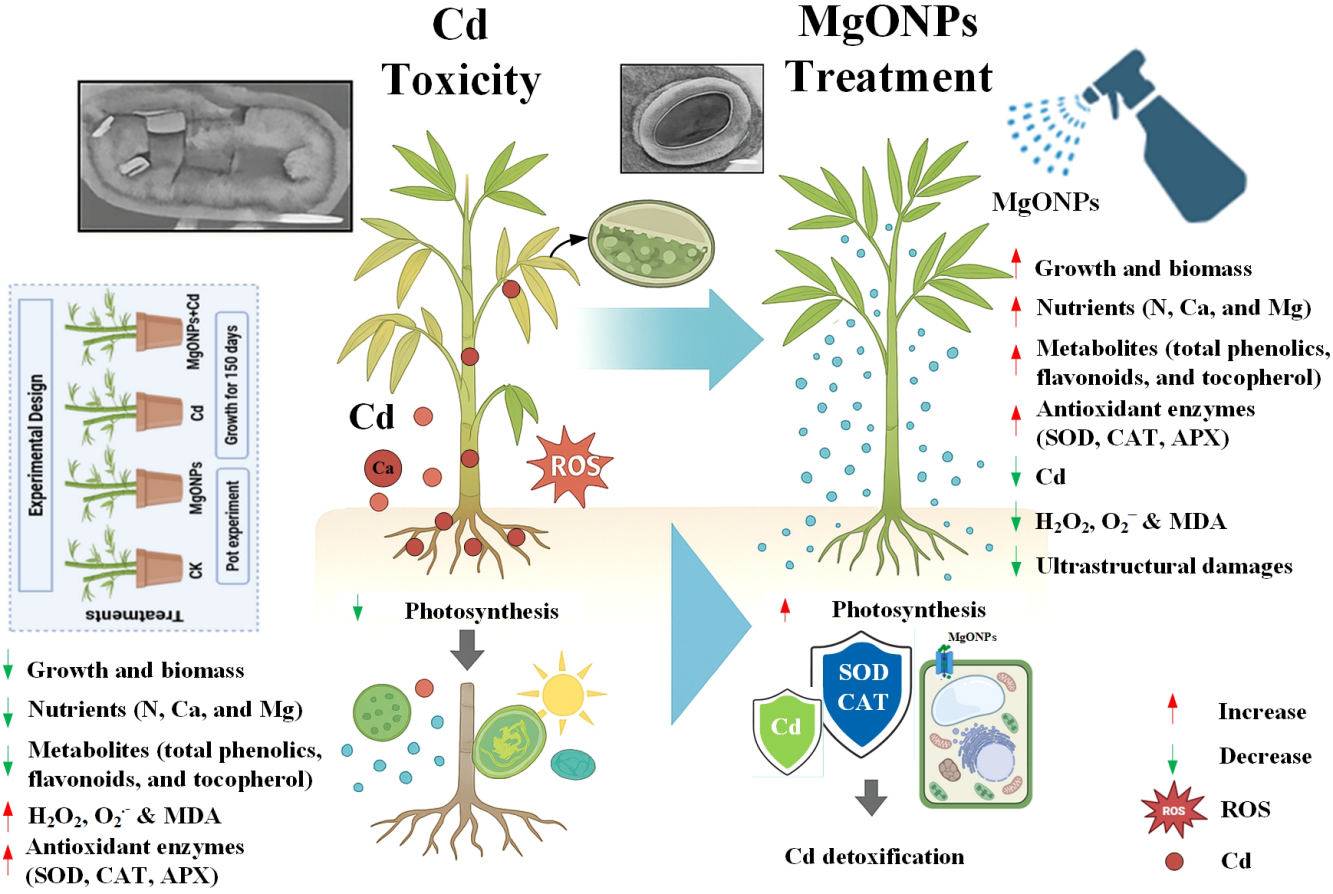
Schematic illustration of MgONPs mitigating Cd stress in bamboo plants.

Based on the current findings, we have drawn a schematic diagram to highlight the protective roles of MgONPs against Cd toxicity in bamboo (**Figure 10**). Our research revealed that MgONPs efficiently enhanced the bamboo tolerance against Cd toxicity. Our findings illustrated that that MgONPs alleviated Cd stress by improving plant growth and biomass production, photosynthetic performance, and nutrients accumulation in plant tissues. Additionally, MgONPs markedly reduced Cd accumulation in stem, shoots, and roots by limiting excess H_2_O_2_ and O_2_^•–^ generation and peroxidation of membrane lipids caused by Cd stress. During Cd stress, MgONPs led to an upregulation in the activities of antioxidant enzymes (APX, CAT, and SOD) and elevated levels of secondary metabolites (total phenolics, flavonoids, tocopherols) highlight their roles in enhancing ROS scavenging and boosting plant resistance to Cd stress. Most importantly, MgONPs protected moso bamboo cells from Cd-induced ultrastructural damage to vital organelles, including the vacuole size, cell wall, chloroplasts, mitochondria, and thylakoid membranes. This study recommends pre-treating bamboo plants with MgONPs as an effective strategy to mitigate Cd phytotoxicity and reduce the related risks to soil-plant and human health. The findings of this study can help design practical applications for MgO nanoparticles as Mg-based nanofertilizers, effectively mitigating soil heavy metal contamination and enhancing bamboo’s tolerance to stress. Further field experiments should emphasize the molecular mechanisms of MgONPs in regulating heavy metal-related genes, soil biological processes, and their practical effectiveness.

## Data Availability

The original contributions presented in the study are included in the article/supplementary material. Further inquiries can be directed to the corresponding authors.
